# Dependency of Non-Small Cell Lung Cancer Cells on Glutamine and Glucose Levels in the Presence of Metformin

**DOI:** 10.5152/eurasianjmed.2026.251018

**Published:** 2026-03-04

**Authors:** Şahika Cıngır Köker, İrem Doğan Turaçlı

**Affiliations:** 1 Department of Medical Biology, Ufuk University Faculty of Medicine, Ankara, Türkiye; 2 Öğretmen Naime Tömek Research Laboratory (ÖNTAL), Ufuk University Faculty of Medicine, Ankara, Türkiye

**Keywords:** Glutamine, KRAS, NSCLC, metformin, proliferation

## Abstract

**Background:**

Metabolic shift is one of the hallmarks of cancer cells. Due to mutations in oncogenes such as Kirsten Rat Sarcoma Viral Oncogene (KRAS), cancer cells can adapt to stress-induced conditions. One of the adaptations that is commonly observed in non-small cell lung cancer (NSCLC) cells is glutaminolysis, where they exhibit high dependency on the presence of glutamine. Metformin is used for its anti-tumor effects, which inhibit mitochondrial complex I. This study aimed to investigate how glucose and glutamine availability affect the proliferation of three KRAS mutant NSCLC cells under metformin pressure.

**Methods:**

Using gene expression datasets, it was observed that glutamine was the second most affected metabolite upon metformin-treated A549 cells. Based on this, several 3-(4,5-dimethyltiazol-2-yl)-2,5-diphenyltetrazolium bromide (MTT) assays were done by using high and low glucose conditions having different concentrations of glutamine at different time points. Moreover, metformin was added to the setup to observe the flexibility of the cancer cells in terms of metabolic switches.

**Results:**

Addition of glutamine resulted in a decrease in metformin’s antiproliferative effect especially in high glucose conditions at later time points. A significantly higher proliferation rate in low glucose conditions compared to high glucose conditions was observed, which is especially pronounced with the addition of glutamine. These observations were supported by the gene expression analysis of the GSE dataset, which revealed upregulation of apoptosis related genes and downregulation of proliferation-related genes in metformin-treated A549 cells.

**Conclusion:**

Taken together, the results highlight the importance of targeting different metabolites and metabolic pathways in cancer therapy.

**Main Points**

- Under energy stress, metformin makes cancer cells more vulnerable when glutamine is scarce, highlighting glutamine’s crucial metabolic role.
- Interestingly, low glucose conditions support the proliferation of cancer cells, shifting their metabolism toward glutamine utilization and oxidative phosphorylation.
- In high glucose and glutamine-depleted conditions, metformin’s effect is increased by disrupting mitochondrial function in glycolytic cancer cells.
- Glutaminolysis appeared as one of the major pathways to balance redox homeostasis in Kirsten Rat Sarcoma Viral Oncogene-mutant cancer cells, making this pathway an attractive therapeutic target.

## Introduction

It is a very well-known fact that tumor cells have different metabolic activity patterns in contrast to normal cells. This occurs due to the changes in their transcriptomic program and signaling pathways, which lead to uncontrolled proliferation, survival under stress conditions, and resistance to apoptosis.^1^ These changes are often driven by oncogenic mutations and epigenetic modifications within the tumor. Due to this fact, cancer cells exhibit heterogeneity inside the tumor, as well as in the tumor microenvironment. The cancer cells within the same environment can exhibit significant metabolic heterogeneity, adapting their energy production and biosynthetic processes in response to local nutrient availability, oxygen tension, and intercellular interactions. Since these kinds of changes are observed across many cancer types, it is represented as “Hallmark of Cancer.”^2^

One of the key adaptations that cancer cells rely on is continuing glycolysis even when oxygen is available, a phenomenon called the “Warburg Effect.” Normal cells shift to glycolysis under low oxygen conditions (hypoxia); however, cancer cells continue glycolysis regardless of the presence of oxygen, which provides them certain advantages. By this way, cancer cells obtain the necessary intermediates that can be used as building blocks during proliferation.^3^ Since the cells divert glycolytic intermediates into anabolic pathways, this negatively affects the conversion of pyruvate to acetyl-CoA, which is necessary for the continuity of the tricarboxylic acid (TCA) cycle.^4^ To balance the reduced flow of glucose-derived carbon into acetyl-CoA and the continuous flow of TCA cycle intermediates for biosynthesis, proliferating cancer cells increase their uptake of the non-essential amino acid, glutamine. Glutamine, together with its metabolic derivatives glutamate and α-ketoglutarate (α-KG), provides nitrogen and carbon that are used for the synthesis of purines, pyrimidines, and amino acids. Glutaminolysis (catabolism of glutamine), together with the carboxylation of pyruvate to oxaloacetate, serves as the major pathways feeding the TCA cycle (anaplerosis).^5^

Due to the mutations or abnormal expressions of oncogenes and tumor suppressor genes, cancer cells can increase their dependency on glutamine. This can be reached by upregulation of glutamine transporters such as ASCT2 (solute carrier family 1 member 5 *[ASCT2]*) and the increased expression of the enzymes involved in glutamine metabolism like glutaminase (GLS1).^6^ When glutamine enters the cell, glutaminolysis takes place which is mediated by GLS1. As a result, glutamate is formed from the catabolism of glutamine, which is then converted to α-KG. This process is vital for cancer cells since they obtain carbons for the citric acid cycle to produce glutathione, nucleotides, and fatty acids. Because cancer cells are highly dependent on glutaminolysis, they are sensitive to the levels of glutamine.^7^

One of the most frequently diagnosed cancer types leading to a significant number of deaths is lung cancer.^8^ Non-small cell lung cancer (NSCLC) is the most common type of lung cancer.^9^ Although there have been many developments in treatment strategies, the 5-year survival rate of advanced NSCLC is still quite low;^10^ therefore, new strategies are in high demand.

The most common genetic changes that occur in lung cancer are the mutations in the Kirsten Rat Sarcoma Viral Oncogene (*KRAS*) gene.^11^ There is a body of evidence in the literature about the link between KRAS mutation and glutamine metabolism. For example, cancer cells having a mutation in KRAS are characterized by increased glycolytic flux and greater dependence on glutamine metabolism. This helps them to maintain redox homeostasis and support anabolic processes.^12^ Moreover, in a study, it was shown that inhibiting glutamine metabolism in pancreatic ductal adenocarcinoma enhanced the sensitivity of the cells to radiotherapy by elevating oxidative stress.^13^

In terms of cell proliferation and survival, glycolytic pathways play major roles. Therefore, targeting these altered metabolic pathways emerged as an alternative therapeutic strategy.^14^ At this point, metformin, the most prescribed drug for the treatment of type II diabetes (TIID), has gained importance due to its effects on cell metabolism.^15^ In addition to its effect on reducing glucose levels, metformin also activates AMP-activated protein kinase (AMPK), the negative regulator of mammalian target of rapamycin, resulting in inhibition of cell proliferation.^16^ On the other hand, metformin exerts its action by inhibiting mitochondrial electron transport chain complex I, which results in downregulation of cytosolic OXPHOS (oxidative phosphorylation). This impairs oxidative phosphorylation, which is essential for tumor growth.^17^ Moreover, several studies showed that metformin is associated with the decreased risk of lung cancer in patients having TIID.^18^

Based on these, it is hypothesized that glucose and glutamine levels differentially affect KRAS mutant NSCLC cell line proliferation. To test this, the cells were treated with varying levels of glutamine in combination with high or low glucose containing conditions. By this way, an opportunity arose to test whether exogenous glutamine could make the cancer cells escape from inhibition of proliferation induced by metformin. The observations indicate that glutamine addition diminishes the antiproliferative effect of metformin, specifically in high glucose conditions at later stages of treatment, potentially compromising metformin’s efficacy.

## Material and Methods

### Gene Expression Data Acquisition and Processing

Public data were retrieved from the NCBI Gene Expression Omnibus (GEO) (https://www.ncbi.nlm.nih.gov/geo/); the GEO Series (GSE146982) was downloaded and analyzed by using the GEO2R tool, a web-based platform that allows users to compare two or more groups of samples within a GEO series. Samples were grouped according to metformin treatment vs. control in A549 vector treated cells. Genes with an adjusted *P*-value < .05 and an absolute log2 fold-change > 1 were considered statistically significant. The Volcano plot was generated using GEO2R. To see the most affected metabolites, the top 250 genes were analyzed in the Enrichr web-based tool.^19^

### Cell Culture

A549, Calu1, and H2009 cells were maintained in high glucose DMEM (Dulbecco’s Modified Eagle Medium) with 10% FBS (fetal bovine serum) at 37°C, 5% CO_2, _and 95% humidity. The cultured cells were passaged to another cell culture flask by using trypsin when they covered 70% of 75 cm^2^ flasks.

### 3-(4,5-Dimethyltiazol-2-yl)-2,5-Diphenyltetrazolium Bromide Assay

5 × 10^3^ A549 and H2009, 6 × 10^3^ Calu1 cells were seeded in 96-well plates. Groups are treated with high glucose (4.5 g/L) with (1% [2 mM] or 2% [4 mM]) or without glutamine or low glucose (1 g/L) with (1% [2 mM] or 2% [4 mM]) or without glutamine, and 10 mM metformin (Merck cat: 317240), unless otherwise stated. At the end of either 24, 48, or 72 hours, MTT (3-(4,5-dimethyltiazol-2-yl)-2,5-diphenyltetrazolium bromide) (Merck, #475989) assay was performed based on the manufacturer’s instructions. Microplate reader (SpectraMax iD3) was used for color absorbance at a wavelength of 570 nm. Results were normalized against the mean measurements from at least 6 replicates that were not exposed to any treatment. Each experiment was repeated twice, and within each experiment all conditions had 3 technical replicates.

**Ethics Committee Approval:** This study did not involve any human participants, animal subjects, or patient data. Only established and commercially available cell lines (A549, Calu-1, and H2009) were used. Therefore, ethics committee approval was not required under the institutional policies or international guidelines. As these cell lines are fully anonymized and not linked to any identifiable individual, they are exempt from ethics review requirements.

**Informed Consent:** Since this study did not involve any human participants or patient data, it is exempt from informed consent.

### Statistical Analysis

GraphPad (Prism 10) was used to plot graphs and for statistical analysis. With OD values, MTT results were plotted and analyzed by using two-way ANOVA with multiple comparison tests (Tukey’s multiple comparison tests).

## Results

**Figure 1.**
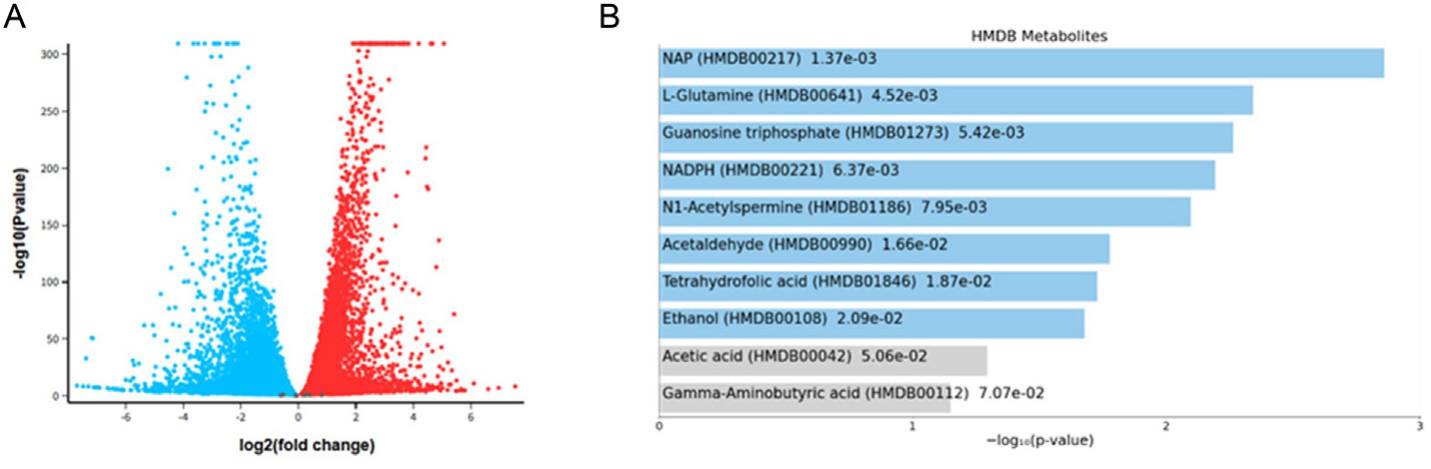
Bioinformatic analysis of GSE146982 data set for A549 cells treated with or without metformin A) Differentially changed genes upon metformin treatment in A549 cells (*P* adj < .05). B) Top 10 metabolites affected by the presence or absence of metformin in A549 cells.

To investigate the transcriptional response to metformin, the publicly available RNA-seq (GSE146982) dataset was used, which included only control vector–transfected A549 cells treated with or without metformin. Subsequently, the top 250 genes were analyzed in a web-based tool, Enrichr,^19^ for gene set enrichment analysis, and glutamine appeared as the second most interactive metabolite in this setup (Figure 1).

**Figure 2.**
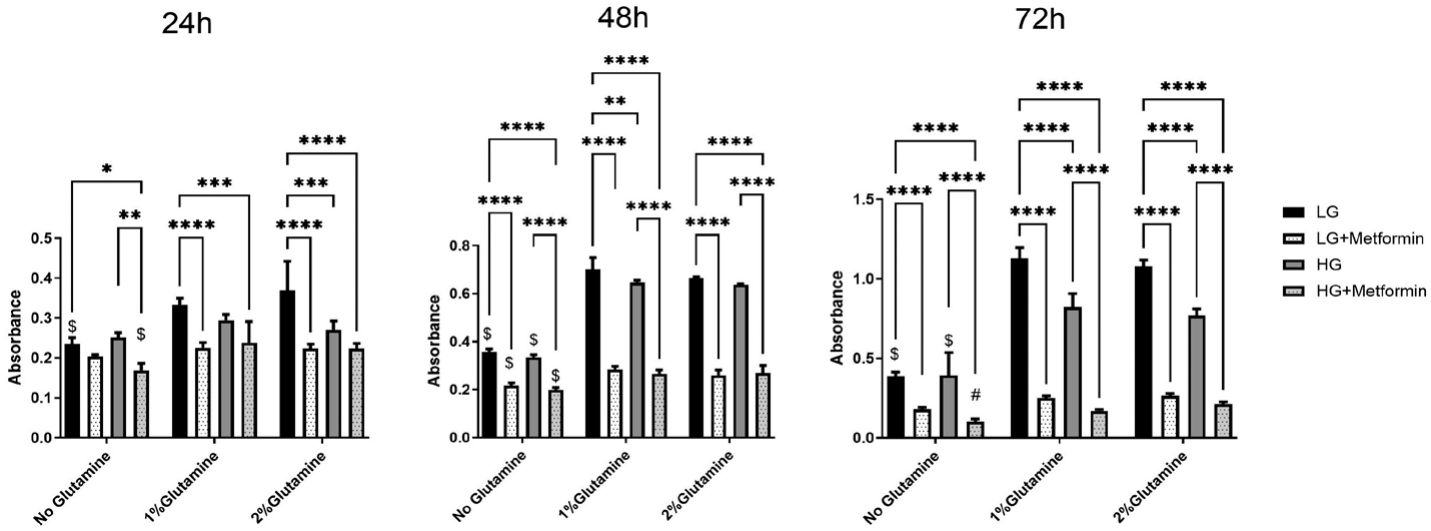
Changes in proliferation rate in low (1 g/L) and high (4.5 g/L) glucose conditions with or without glutamine upon 10 mM metformin treatment in A549 cells. * *P* < .05, **P < .01, ***P < .001, ****P < .0001. $ shows the comparison between no glutamine vs. 1% (2 mM) glutamine and 2% (4 mM) glutamine. # shows the comparison between no glutamine vs. only 2% glutamine.

**Figure 3.**
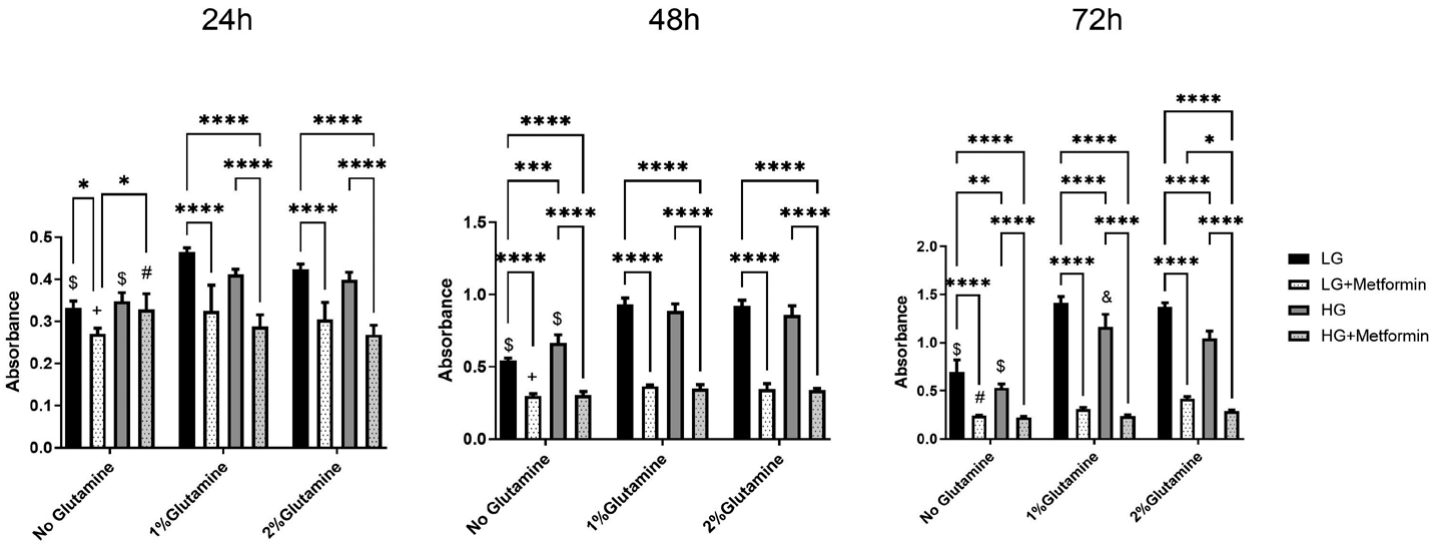
Changes in proliferation rate in low (1 g/L) and high (4.5 g/L) glucose conditions with or without glutamine (2 mM or 4 mM) upon 10 mM metformin treatment in Calu1 cells. *P < .05, **P < .01, ***P < .001, ****P < .0001. (+) shows the comparison between no glutamine vs. only 1% glutamine; (#) shows the comparison between no glutamine vs. only 2% glutamine; ($) shows the comparison between no glutamine vs. both 1% glutamine and 2% glutamine within the same treatments. (&) shows 1% glutamine vs. 2% glutamine.

**Figure 4.**
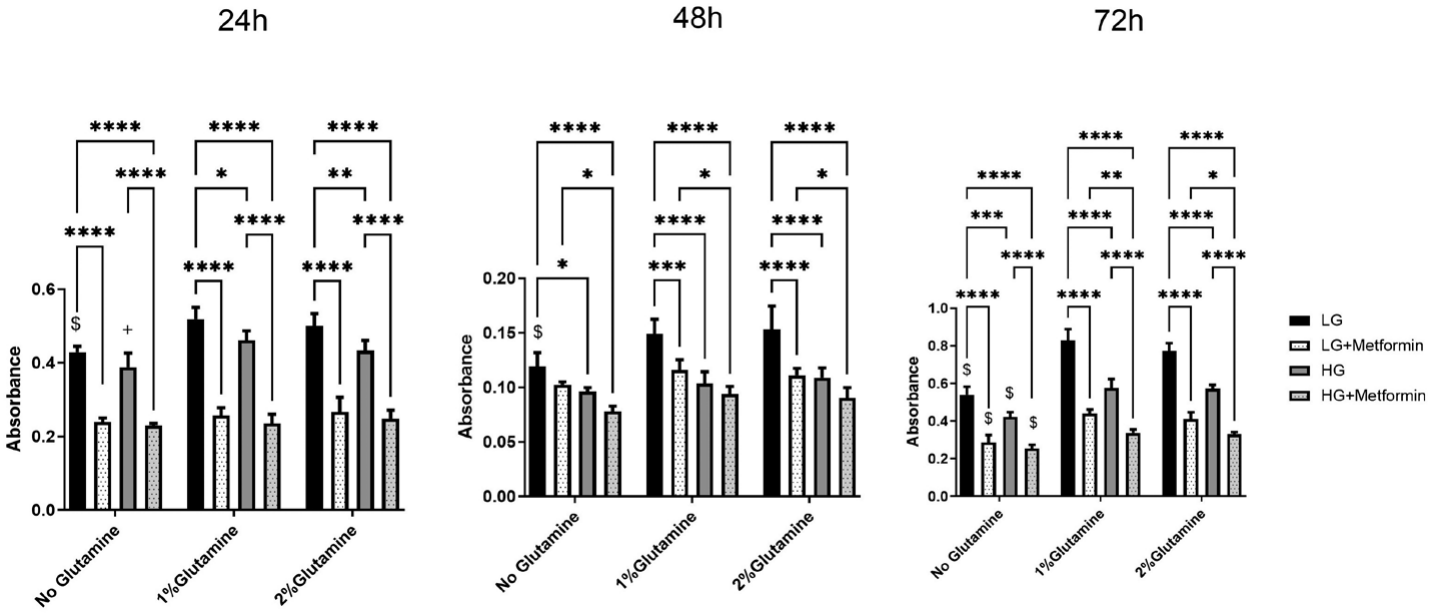
Changes in proliferation rate in low (1 g/L) and high (4.5 g/L) glucose conditions with or without glutamine (2 mM or 4 mM) upon 10 mM metformin treatment in H2009 cells. *P < .05, **P < .01, ***P < .001, ****P < .0001. (+) shows the comparison between no glutamine vs. only 1% glutamine; (#) shows the comparison between no glutamine vs. only 2% glutamine; ($) shows the comparison between no glutamine vs. both 1% glutamine and 2% glutamine within the same treatments.

Based on these findings, the effect of metformin in lung cancer cells in the presence and absence of glutamine was tested. In A549 cells, at all 3 time points, a significantly higher proliferation rate in low glucose conditions upon 1% or 2% glutamine addition (see $ on the graph) was observed (Figure 2). In contrast, this type of increase in proliferation in high glucose conditions upon 1% or 2% glutamine addition was observed after 48 and 72 hours (see $). Compared to the effects resulting from glutamine addition, metformin addition acted similarly in both low glucose and high glucose conditions at all time points. However, metformin was less effective after the addition of 1% and 2% glutamine at 24 and 48 hours in high glucose conditions. After 72 hours, metformin was less effective only in high glucose conditions with 2% glutamine compared to no glutamine group. However, in low glucose conditions, there was no effect of metformin after 24 hours both in the presence and absence of glutamine. After 48 hours, the effect of metformin was abolished in the presence of glutamine (both 1% and 2%). Interestingly, this effect disappeared after 72 hours, which might result from metabolic adaptation. Moreover, unlike Calu1 (Figure 3) and H2009 (Figure 4) cells, metformin reduced the proliferation of A549 cells both in the presence of high and low glucose conditions to a similar extent (Figure 2).

Like A549 cells, significantly higher proliferation rates in low glucose conditions both in the presence and absence of glutamine at all time points compared to high glucose conditions in Calu1 cells was observed (Figure 3). Moreover, the addition of 1% or 2% glutamine increased this rate even more. Like A549 cells, at 24 hours and 48 hours, metformin’s effect was abolished with the addition of 1% glutamine in low glucose conditions (+ on bar chart). But after 72 hours, only the 2% addition of glutamine eliminated metformin’s anti-proliferative effect in low glucose conditions (# on bar chart). Similarly, a higher proliferation rate in high glucose conditions in the presence of glutamine at all time points compared to no glutamine-high glucose containing groups was observed. Interestingly, while metformin reduces the proliferation of Calu1 cells in high glucose and 2% glutamine containing groups at 24 hours, this kind of effect disappeared at 48 and 72 hours; namely, the addition of glutamine, either 1% or 2%, did not prevent the anti-proliferative effect of metformin.

Like A549 and Calu1, H2009 cells also proliferated more in low glucose conditions; this rate was pronounced with both 1% and 2% glutamine addition (Figure 4). The addition of metformin in low glucose conditions did not differ between glutamine additions for 24 hours and 48 hours; rather, after 72 hours, it abolished metformin’s effect. The addition of 1% glutamine significantly increased proliferation in high glucose conditions compared to the one which did not have any glutamine for 24 hours. Interestingly, there was no difference in proliferation rates with the addition of glutamine and/or metformin in high glucose conditions at 48 hours, but still, the proliferation rates in high glucose conditions were lower compared to low glucose conditions. Metformin was again not effective in high glucose conditions in terms of reducing proliferation with the addition of glutamine at 24 and 48 hours. Like Calu1 cells and unlike A549 cells, one can observe the anti-proliferative effect of metformin in glutamine containing high glucose conditions at 24 hours, but this effect disappeared at 72 hours.

**Figure 5.**
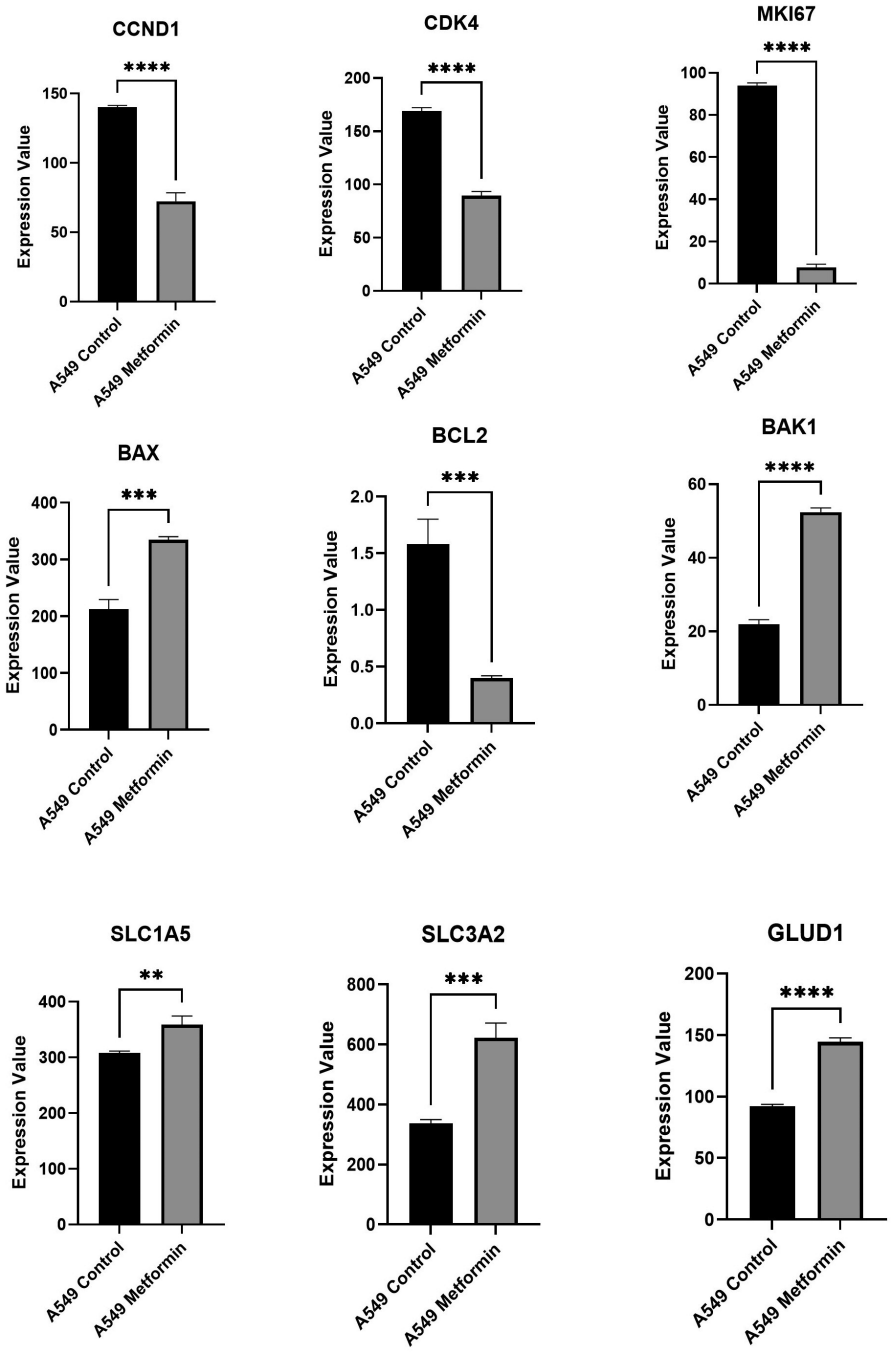
The expression pattern of the genes related to proliferation (CCND1, CDK4 and MKI67), apoptosis (BAX, BCL2 and BAK1), and glutamine metabolism (SLC1A5, SLC3A2 and GLUD1). Comparison is done Control vs. Metformin. *P < .05, **P < .01, ***P < .001, ****P < .0001.

Since decreased proliferation upon metformin treatment was observed, the expression of the genes related to proliferation, apoptosis, and glutamine metabolism in the dataset that were checked in the beginning (GSE146982) were examined. Consistent with the MTT data, it was found that the expression of proliferation related genes (CCND1 [Cyclin D1], CDK4 [Cyclin-dependent kinase 4], MKI67 [Marker of proliferation Ki-67]) was decreased, whereas the expression of the pro-apoptotic genes (BAX [BCL2-associated X protein], (BAK1 [BCL2 antagonist/killer 1]) was increased, and the anti-apoptotic gene (BCL2 [BCL2 apoptosis regulator *(B-cell lymphoma 2)*]) was downregulated (Figure 5). Moreover, the major genes participating in glutamine transport such as the key glutamine transporters SLC1A5 [Solute carrier family 1 member 5 *(ASCT2)*] and SLC3A2 [Solute carrier family 3 member 2] increased upon metformin treatment. This aligns with the metabolic adaptation of KRAS mutant NSCLC which exhibits high dependency on glutamine to feed the anabolic reactions as well as TCA cycle flow. The expression of the enzyme which converts glutamate to α-KG and GLUD1 was also increased upon metformin treatment (Figure 5).

## Discussion

Glutamine metabolism is one of the major pathways playing important roles in cancer cell metabolism. Glutamine serves as a crucial anaplerotic substrate, replenishing the TCA cycle and supporting ATP production, redox homeostasis, and biosynthesis. In contrast, metformin acts to reduce ATP production by inhibiting mitochondrial complex I, activating AMPK, and subsequently suppressing the mTOR pathway, all of which contribute to reduced cell proliferation.^20^

Metabolic plasticity, which is one of the hallmarks of cancer cells, allows cancer cells to adapt to microenvironmental changes; hence, it increases their survival. High glycolytic flux is observed in cancer cells despite the presence of intact mitochondrial respiration, which presents one of the plasticities that these cells exhibit.^21^ Despite being less efficient than OXPHOS in terms of ATP yield, it is still favored by cancer cells due to its capacity to produce intermediates needed for biosynthetic pathways, such as building blocks for nucleic acids, proteins, and lipids. Moreover, this kind of metabolic shift also creates an acidic environment through lactate production, which eventually contributes to tumor cell invasion as well as suppressing immune responses.^22^

By being largely mutated in tumors such as colon cancer and NSCLC, KRAS turns on the proteins and locks them in an “on” state. This leads to constitutive activation of the MAPK and PI3K/mTOR pathways, which results in an increased proliferation rate together with resistance to apoptotic signals.^23^ Moreover, KRAS activation favors TCA anaplerosis via glutamine, reducing the cycle’s reliance on glycolytic carbon. In other words, mutant KRAS drives metabolic rewiring in cancer cells.^24^ It is also known that glutaminase activity is linked to the RAF-MEK-ERK signaling pathway and that EGF-driven MEK-ERK activation results in increased glutaminase activity, while the MEK1/2 inhibitor U0126 suppresses it.^5^ Moreover, the KRAS signaling cascade also increases the expression of the genes encoding the transporters and enzymes such as SLC1A5 and GLS1.^25^ The increased activity of glutaminase results in the conversion of glutamine to glutamate and α-KG. In this way, TCA anaplerosis, aspartate and nucleotide synthesis, as well as redox balance are sustained.^26^ Since metformin exerts its effect by inhibiting complex I, limiting mitochondrial NAD^+^ regeneration and aspartate production, KRAS-mutant cells become especially reliant on glutamine-derived anaplerosis to keep biosynthesis going. Hence, the presence of glutamine blunts metformin’s effect; conversely, glutamine deprivation intensifies metformin sensitivity.^27^Despite the observed fluctuations between time points, the cells have tendency to be more sensitive to metformin under glutamine-deprived conditions, indicating that the lack of glutamine increases the dependency of cancer cells on this metabolic pathway. Although quantitative statistical analyses are not provided here, graphical trends clearly suggest enhanced vulnerability to metformin when glutamine is limited.

Interestingly, under low-glucose conditions, increased proliferation, which may initially seem counterintuitive was observed. This phenomenon is likely explained by a metabolic shift in which cancer cells upregulate glutamine metabolism as a compensatory mechanism in response to limited glucose availability. This is supported by the glutamine supplementation data: cells cultured without glutamine showed markedly reduced proliferation, while supplementation with 1% glutamine led to a nearly 2-fold increase in proliferation at 48 hours. Notably, increasing glutamine concentration to 2% did not further enhance proliferation, suggesting that 1% glutamine is sufficient to restore metabolic function and support growth under these conditions.

Moreover, cells in low glucose conditions may undergo a metabolic switch toward oxidative phosphorylation. Such a switch may activate survival pathways and allow cells to sustain growth even in the face of reduced glycolytic flux, which shows a highly dynamic phase of metabolic reprogramming.^28^ This shift could also make them relatively more resistant to metformin, whose primary mechanism involves targeting mitochondrial respiration. In contrast, under high glucose conditions, cancer cells are more glycolytic and less reliant on oxidative phosphorylation. As such, metformin is more effective in high glucose conditions, particularly when combined with glutamine deprivation, as glycolytic cells are more susceptible to energy stress induced by metformin.

The major source of ROS (reactive oxygen species) is mitochondria and elevated levels of ROS result in cell death of KRAS mutant cells.^29^ To prevent this, KRAS mutant cells rely mostly on glutaminolysis even in the presence of oxygen. By this way, they counteract the damaging effects of ROS which even makes them resistant to chemotherapy. These findings clearly emphasize the critical role of altered glutamine and energy related pathways in terms of regulating cell proliferation and survival in KRAS mutant cells.

Different response patterns across A549 (KRAS-G12S), Calu-1 (KRAS-G12C), and H2009 (KRAS-G12A) observed in this study support the idea of KRAS allele-specific effects on signaling pathways.^30^ As shown in the literature, Calu-1 (KRAS-G12C) cells exhibited a different response to the MEK inhibitor compared to A549 (KRAS-G12S) and H2009 (KRAS-G12A) in terms of increasing CSNK2A1/CK2 function, which determines the sensitivity to the MEK inhibitor.^31^ In addition to this, the changes mostly seen in clinical samples, such as in TP53, STK11/LKB1, and KEAP, can accompany this situation, which can redirect metabolic routes to enhance allele-specific changes, such as increased reliance on glutamine metabolism and reshaped mitochondrial pathways, which are consistent with the observation.^32^ Another study demonstrated that metformin’s antitumor effects arise from inhibiting glutaminase activity and suppressing autophagy in breast and cervical cancer cell lines. The reduction in autophagy is accompanied by stronger BECN1–BCL2 interaction and increased apoptotic cell death. This provides a mechanistic rationale for metformin’s anti-tumor effects, which are mediated through the suppression of glutaminase activity and autophagic processes.^33^ Also, another study showed that glutamine deprivation or inhibition using a glutaminase inhibitor reduced cell growth and triggered apoptosis in SKM-1 leukemia cells. On the other hand, metformin also inhibits SKM-1 proliferation and enhances apoptosis. Furthermore, glutamine deprivation combined with metformin treatment enhanced these cells’ sensitivity to cytarabine.^34^ So, the combination of metformin and strategies targeting glutamine metabolism reflects a new perspective and offers a promising therapeutic approach for acute myeloid leukemia, and preclinical studies also demonstrate efficacy in KRAS-mutant ovarian cancer, where CB-839 plus metformin/phenformin synergistically induces apoptosis and reduces tumor growth.^35^

Understanding and modulating the metabolic reprogramming of the cell, such as glutamine metabolism under energy stress, could enhance treatment efficacy, particularly in metabolically adaptive cancer cells. These findings highlight the context-dependent metabolic plasticity of cancer cells and emphasize the importance of both glucose and glutamine availability in modulating the efficacy of metabolic inhibitors like metformin. The interplay between nutrient levels and metabolic inhibitors may inform future strategies for combination therapies targeting cancer metabolism.

This study is limited to in vitro models and uses proliferation assays as indirect readouts of metabolic state. Respiration/flux analyses or in vivo validation were not performed, and the 3 KRAS-mutant lines cannot capture the full patient heterogeneity. Future studies will use more than 3 independent biological replicates per condition, measure OCR and ECAR, apply 13C tracing to quantify fluxes, include broader KRAS-mutant and KRAS-WT panels, and validate findings in xenograft or organoid models.

## Data Availability

The data that support the findings of this study are available on request from the corresponding author.
